# Ovarian Clear Cell Carcinoma with Functioning Stroma and Associated Endometrial Intraepithelial Neoplasia

**DOI:** 10.1155/2022/5538390

**Published:** 2022-07-06

**Authors:** Fabian Desimpel, Jean-Christophe Noël, Laurine Verset, Nicolas Sirtaine, Frédéric Buxant

**Affiliations:** ^1^Department of Obstetrics and Gynecology, Iris South Hospital, Brussels, Belgium; ^2^Department of Pathology, Erasme University Hospital, Free University of Brussels, Belgium; ^3^Department of Pathology, Institut Jules Bordet, Free University of Brussels, Belgium

## Abstract

We report the case of a 79-year-old woman with a large pelvic mass and postmenopausal bleeding, associated with hyperestrogenism. A pelvic MRI shows the presence of a large mass of 12.6 cm originating from the right ovary without signs of metastasis. A total abdominal hysterectomy with unilateral salpingooophorectomy was performed, knowing the patient underwent a left salpingooophorectomy decades ago. The pathological findings showed an ovarian clear cell carcinoma (pT1A) with associated endometrial intraepithelial neoplasia. There is convincing evidence that the production of estrogen is located in the activated ovarian stroma. This supports the view that functioning stroma of ovarian cancer can lead to hyperestrogenism and eventually endometrial cancer.

## 1. Introduction

Ovarian clear cell carcinoma (OCCC) is a subtype of epithelial ovarian carcinoma (EOC) [1] which is believed to develop from ovarian endometrial cysts as a direct precursor lesion [2, 3]. It occurs in 5-25% of epithelial ovarian cancers with an increased prevalence in Eastern-Asian women. Patients with OCCC tend to present at earlier stages than patients with high-grade serous tumors, and when compared to stage-matched controls, patients with early-stage OCCC may have a better prognosis. However in advanced stages (1C-IV), it is associated with relatively poor prognosis due to the lack of chemosensitivity to platinum-based chemotherapy [4, 5]. The stroma of epithelial ovarian carcinoma sometimes consists of a specialized ovarian stroma with endocrine function, called a functioning stroma. The origin of this functioning stroma, its histogenetic mechanism, and relationship to prognosis remain to be clarified [6]. Functional ovarian neoplasms have unique clinical manifestations related to hormone overproduction and may give rise to a broad spectrum of clinical syndromes. Hyperestrogenism due to functioning ovarian stroma has been reported typically in sex cord-stromal tumors such as granulosa cell tumors and to a lesser extent some Sertoli-stromal cell tumors. This hyperestrogenism can result in thickening of the endometrium or abnormal maturation of cervical smears, particularly in postmenopausal women [7]. In the literature, cases of clear cell carcinoma of the ovary with associated hyperestrogenism produced by functional stroma have been described [8–10].

## 2. Case Report

A 79-year-old female was referred to the gynecology service for diagnostic workup of postmenopausal bleeding since 3 weeks and a large pelvic mass. Reproductive history was significant for 3 first trimester pregnancy losses and 1 ectopic pregnancy at the age of 29, for which she underwent an emergency left salpingooophorectomy. Thus nulliparous, she is menopausal without ever using hormone replacement therapy. Her past family history was unremarkable, and she denied ever using tobacco. A pelvic examination identified a very large mass occupying the whole pelvis. Pelvic and abdominal ultrasound showed a cystic mass with a solid component, measuring >12 cm. Complete serum blood count was unremarkable, and serum tumor marker levels were cancer antigen 125 (CA125) at 69 kU/L (reference range < 35 kU/L), Human Epididymal Protein 4 (HE4) at 439 pmol/L (reference range < 104 pmol/L), and carcinoembryonic antigen (CEA) at 1.6 *μ*g/L (reference range < 5.2 *μ*g/L). Although there was no indication to measure the serum estradiol level, the general practitioner measured it at 134 ng/L (reference range < 50 ng/L), as the only hormone measured. Pelvic magnetic resonance imaging (MRI) confirmed the presence of a large mass of 12.6 cm with cystic and solid components, originating from the right ovary. There were no radiological signs of pathological lymph nodes, ascites, or peritoneal carcinomatosis. Computed and position emission tomography showed fluorodeoxyglucose uptake only in the pelvic tumor.

**Figure 1:**
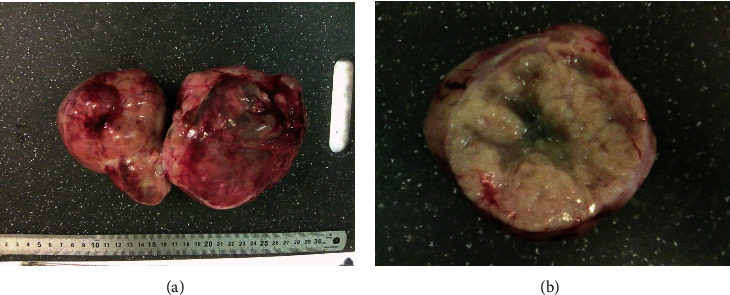
Macroscopic findings of (a) complete resected specimen and (b) cross-section of the right ovary with firm and fibrous yellow-white tumor.

**Figure 2:**
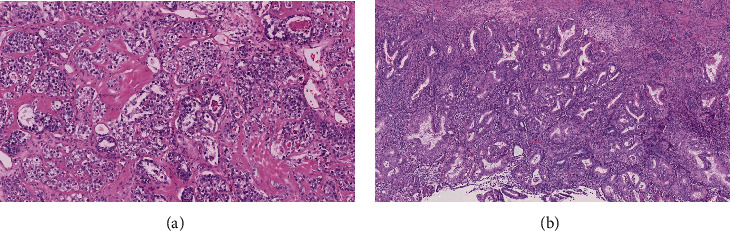
Microscopic findings of (a) the clear cell carcinoma and (b) the associated EIN (hematoxylin and eosin staining (H&E), ×40).

Since no metastatis was suspected, a clinical diagnosis of ovarian cancer stage 1A was made. A laparoscopic staging with adhesiolysis was performed, confirming a tumor limited to the right ovary. There were no signs of a left ovary in accordance with her surgical history, and the uterus appeared to have no remarkable changes. After peritoneal lavage, the procedure was completed by conversion to laparotomy and a total abdominal hysterectomy with unilateral salpingooophorectomy was performed. No complications occurred during surgery, and the postoperative course was uneventful. Pathological findings showed an ovarian clear cell carcinoma (pT1A) with associated endometrial intraepithelial neoplasia (EIN) and mullerianosis and a negative peritoneal washing cytology (Figures 1 and 2).

**Figure 3:**
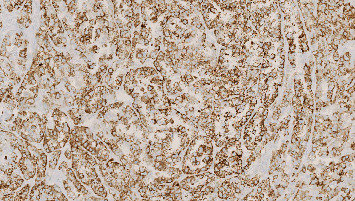
Immunohystochemical staining of the stromal part-calretinin (×100).

Immunohistochemical analysis of the right ovary showed that P405S and Napsin A were positive in the carcinomatous part. Moreover, inhibin alpha and calretinin were positive in the stromal part (Figure 3). Genetic analysis revealed a phosphatidylinositol-4,5-bisphosphate 3-kinase catalytic subunit alpha (PIK3CA) mutation, one of the most frequent specific gene alterations in ovarian clear cell carcinoma [11]. Upon follow-up, estradiol levels returned to zero, and eight months after surgery, no sign of recurrence was noted.

## 3. Discussion

Unopposed estrogen leads to endometrial stimulation, which can occasionally result in endometrial hyperplasia and carcinoma. In postmenopausal women, exogenous estrogen may be from hormone replacement therapy or from other nonprescribed sources such as herbal remedies. Less commonly, women with a hormonally active ovarian neoplasm present with endometrial symptoms. Regardless of source, increased estrogen not counterbalanced by progesterone can lead to a field effect of benign hyperplasia, which can lead to the development of EIN [12].

The patient in our case consulted her general practitioner because of postmenopausal bleeding since 3 weeks, being thus the first symptom leading to the diagnosis of an ovarian clear cell carcinoma. There is convincing evidence that the observed hyperestrogenism was caused by the functioning ovarian stroma, since there was expression of markers of sex-steroid differentiation and steroidogenesis, namely, inhibin alpha and calretinin in the stroma [13]. In the present study, no signs of estrogen production in the malignant epithelium itself were observed. This patient had no hormone replacement therapy, and a concomitant production of estrogen by the contralateral ovary can be excluded since she underwent a left salpingooophorectomy. Moreover, the estrogen levels returned to zero in the immediate postoperative period.

Therefore, this case supports the view that the stroma surrounding epithelial tumors in the ovary are activated to elaborate steroid hormone, which may have stimulated endometrial neoplastic growth. The exact mechanism by which the epithelial cancer cells stimulate the surrounding ovarian stromal cells is yet to be elaborated. Since cases of clear cell carcinoma of the ovary with associated hyperestrogenism have been described before and hyperestrogenism can lead to endometrial hyperplasia and carcinoma, it is not surprising that postmenopausal bleeding and EIN can be present in cases of clear cell carcinoma of the ovary. Since we did not find other cases in literature with this association, we present this case in which postmenopausal bleeding and EIN were caused by the functional stroma of clear cell carcinoma of the ovary.

## 4. Conclusion

We report the case of a 79-year-old woman with a large pelvic mass and postmenopausal bleeding, associated with hyperestrogenism in the absence of hormonal treatment. A pelvic MRI shows the presence of a huge mass of 12.6 cm originating from the right ovary without signs of metastasis. A total abdominal hysterectomy with unilateral salpingooophorectomy was performed. The pathological findings showed an ovarian clear cell carcinoma (pT1A) with associated endometrial intraepithelial neoplasia. Estrogen levels returned to zero in the immediate postoperative period.
